# A Decoding Scheme for Incomplete Motor Imagery EEG With Deep Belief Network

**DOI:** 10.3389/fnins.2018.00680

**Published:** 2018-09-28

**Authors:** Yaqi Chu, Xingang Zhao, Yijun Zou, Weiliang Xu, Jianda Han, Yiwen Zhao

**Affiliations:** ^1^State Key Laboratory of Robotics, Shenyang Institute of Automation, Chinese Academy of Sciences, Shenyang, China; ^2^Institutes for Robotics and Intelligent Manufacturing, Chinese Academy of Sciences, Shenyang, China; ^3^University of Chinese Academy of Sciences, Beijing, China; ^4^Department of Mechanical Engineering, University of Auckland, Auckland, New Zealand

**Keywords:** brain-computer interface, decoding scheme, incomplete motor imagery EEG, power spectral density, deep belief network

## Abstract

High accuracy decoding of electroencephalogram (EEG) signal is still a major challenge that can hardly be solved in the design of an effective motor imagery-based brain-computer interface (BCI), especially when the signal contains various extreme artifacts and outliers arose from data loss. The conventional process to avoid such cases is to directly reject the entire severely contaminated EEG segments, which leads to a drawback that the BCI has no decoding results during that certain period. In this study, a novel decoding scheme based on the combination of Lomb-Scargle periodogram (LSP) and deep belief network (DBN) was proposed to recognize the incomplete motor imagery EEG. Particularly, instead of discarding the entire segment, two forms of data removal were adopted to eliminate the EEG portions with extreme artifacts and data loss. The LSP was utilized to steadily extract the power spectral density (PSD) features from the incomplete EEG constructed by the remaining portions. A DBN structure based on the restricted Boltzmann machine (RBM) was exploited and optimized to perform the classification task. Various comparative experiments were conducted and evaluated on simulated signal and real incomplete motor imagery EEG, including the comparison of three PSD extraction methods (fast Fourier transform, Welch and LSP) and two classifiers (DBN and support vector machine, SVM). The results demonstrate that the LSP can estimate relative robust PSD features and the proposed scheme can significantly improve the decoding performance for the incomplete motor imagery EEG. This scheme can provide an alternative decoding solution for the motor imagery EEG contaminated by extreme artifacts and data loss. It can be beneficial to promote the stability, smoothness and maintain consecutive outputs without interruption for a BCI system that is suitable for the online and long-term application.

## Introduction

The emergent brain-computer interface (BCI) technology allows individuals with severe neuromuscular related locomotive disabilities to directly use their brain to operate or communicate with external peripherals and environments (Daly and Wolpaw, 2008; McFarland and Wolpaw, 2011). Namely, the BCI system provides an alternative interface bridge which can bypass the conventional motor neural pathways and map brain intentions to relative control commands (Ortiz-Rosario and Adeli, 2013). Brain activity can be characterized by various signal modalities, such as invasive ElectroCorticoGraphy (ECoG) (Miller et al., 2010; Hiremath et al., 2015), non-invasive electroencephalogram (EEG) (Lazarou et al., 2018), the functional Magnetic Resonance Imaging (fMRI) (Cohen et al., 2014), and the functional Near-Infrared Spectroscopy (fNIRS) (Naseer and Hong, 2015). Due to its manageability, easy capture, high time resolution and relative cost effectiveness, the EEG signal has been widely adopted for substantial BCI applications, such as remote quadcopter control (Lin and Jiang, 2015), motion rehabilitation (Xu et al., 2011; Zhao et al., 2016), biometric authentication (Palaniappan, 2008), and emotions prediction (Padilla-Buritica et al., 2016). Currently, the electrophysiological brain patterns used in EEG-based BCI systems are mainly Steady-State Visual Evoked Potentials (SSVEPs) (Chen et al., 2015; Zhang et al., 2015; Zhao et al., 2016; Nakanishi et al., 2018), P300 (Cavrini et al., 2016), sensorimotor rhythms (SMRs) (Yuan and He, 2014; He et al., 2015), and motion-related cortical potential (MRCP, one kind of a slow cortical potential) (Karimi et al., 2017). Compared to other patterns, the SMRs-based BCI is more flexible and suitable for practical applications due to the spontaneous EEG signals, which are generated by individuals voluntarily without any external stimuli.

The SMRs are derived from the motor imagery EEG, which evoked by mentally imaging the movements of limbs without actual actions (Yuan and He, 2014). The underlying neurophysiological phenomena are event-related synchronization (ERS) and event-related desynchronization (ERD) in the SMRs, which are induced simultaneously by an exogenous event. The variability of ERS/ERD intensity or power in particular frequency bands can be utilized to distinguish the different motor imagery EEG signals (Pfurtscheller et al., 2006; Koo et al., 2015). Some remarkable SMRs-based BCI systems for motor imagery classification have been created and applied in wheelchair control (Li et al., 2013), objects control in 2D (Ma et al., 2017) or 3D space (LaFleur et al., 2013), and robotic arm control (Xu et al., 2011; Meng et al., 2016). However, there are still various challenges faced in the establishment of efficient SMRs-based BCI systems, such as fewer recognizable motor types or states, apparently lower recognition rate, and longer training time (Yuan and He, 2014; He et al., 2015). In addition, due to the volume conduction effect of scalp and skull, the EEG is a non-stationary and non-linear dynamic signal with low signal-to-noise ratio and vulnerable to be interfered or submerged by complex background artifacts, which makes it really challenging to accurately decode various motor imagery tasks (Blankertz et al., 2011). Consequently, the crucial issue that needs to be solved is how to improve the decoding performance of the SMRs-based BCI in the condition of various artifacts.

The artifacts affecting the quality of motor imagery EEG mainly contain electrooculography (EOG), electromyography (EMG) and electrical line interference. Traditionally, a variety of filters can be available to alleviate or even eliminate electrical line interference and some high frequency noises, like EMG (35 Hz above). In the past researches, many typical attempts have been proposed to reduce EOG, such as filter-based method (Shoker et al., 2005), independent component analysis (ICA) (Lindsen and Bhattacharya, 2010) and discrete wavelet transform (DWT) (Peng et al., 2013). However, these methods can cause the loss of some useful EEG components. And the procedures for manual parameter tuning are needed to obtain optimal performance of these methods. Moreover, they generally fail in the case of the EEG contains extreme noises. Otherwise, the EEG signals could be accidentally overwritten or lost caused by hardware or system malfunctions during recording periods. For the above cases, good decoding performance for SMRs-based BCI systems could still hardly be achieved. One intuitive and helpless solution to avoid such extreme artifacts and data loss is usually to reject the entire severely disturbed EEG segments. Consequently, this raises some defects including no decoding results during certain period, additional EEG rejection process and increased BCI training time. Furthermore, from a practical perspective, consecutive and smooth recognition of SMRs-based BCI systems is extremely necessary for the online and long-term application. This requires that the BCI system can continuously decode brain signals without any interruption. If entire EEG segments are discarded due to extreme artifacts or data loss, the BCI system cannot obtain the decoding results during the corresponding time slice. Hence, it is very important to decode incomplete motor imagery EEG for SMRs-based BCI systems in the condition of extreme artifacts and data loss. Currently, only few studies have been conducted to solve the decoding performance from the incomplete EEG signals. Zhang et al. applied a Bayesian tensor factorization based method to find the underlying low-rank EEG tensor from incomplete EEG signals and improve the decoding accuracy with robustness after artifacts and outliers removal (Zhang et al., 2016). Cui et al. used a fully Bayesian CP factorization for incomplete tensors method to analyze and classify incomplete EEG signals with different data missing ratios (Cui et al., 2016). However, such decoding methods for incomplete EEG need complicated matrix and tensor computations, which are not efficient for an online BCI application. Moreover, the classification accuracies obtained by these methods need further improvement.

In this paper, to improve the decoding performance for incomplete motor imagery EEG and satisfying the needs of smooth operation for the BCI system, a novel decoding scheme composed of Lomb-Scargle periodogram (LSP) for feature extraction and deep belief network (DBN) for classification was proposed. Instead of rejecting the entire EEG segment, the portions that affected by extreme artifacts or data loss were directly removed and the remaining portions were used to construct the incomplete motor imagery EEG signals in this study. Generally, the most robust and representative feature for the contents of different motor imageries is spectral power in particular bands of ERS/ERD (Pfurtscheller et al., 2006). The conventional fast Fourier transform (FFT) or Welch periodogram can be available to estimate the spectral power features for the intact motor imagery EEG. Nevertheless, these spectral analysis methods cannot work well for the non-uniformly sampled signals (Stoica et al., 2009), such as incomplete motor imagery EEG signals. The LSP method can handle signals that have been sampled non-uniformly or have missing data points (Stoica et al., 2009; Stankovic et al., 2014) and is suitable for processing incomplete signals. Hence, the LSP method was adopted to extract major spectral power features from the incomplete motor imagery EEG signals in this study. A DBN structure based on the restricted Boltzmann machines (RBM) was exploited and optimized to learn different motor imagery EEG classes. The proposed scheme may offer the following advantages: (a) It can provide comparable decoding performance for the incomplete motor imagery EEG with different proportion of data removal; (b) The extracted spectral power features are more robust for the representation of the incomplete motor imagery EEG; (c) It is applicable to consecutive and smooth operation without any disruption for the online BCI system.

The remaining parts of this paper are organized as follows. The overall systematic framework of decoding scheme for incomplete motor imagery EEG is introduced in section Overall Decoding Scheme Framework. Accordingly, section EEG Processing Pipeline describes the EEG signal processing pipeline in detail, including artifacts and data loss preprocessing, spectral features extraction and DBN classifier construction. The motor imagery experiments and datasets are presented in section Motor Imagery Experimental Paradigm and Datasets. Some experimental comparison results and discussions are given in section Experimental Results and Discussions. Finally, section Conclusions and Future Works gives the conclusions and ideas for future works.

## Overall decoding scheme framework

**Figure 1:**
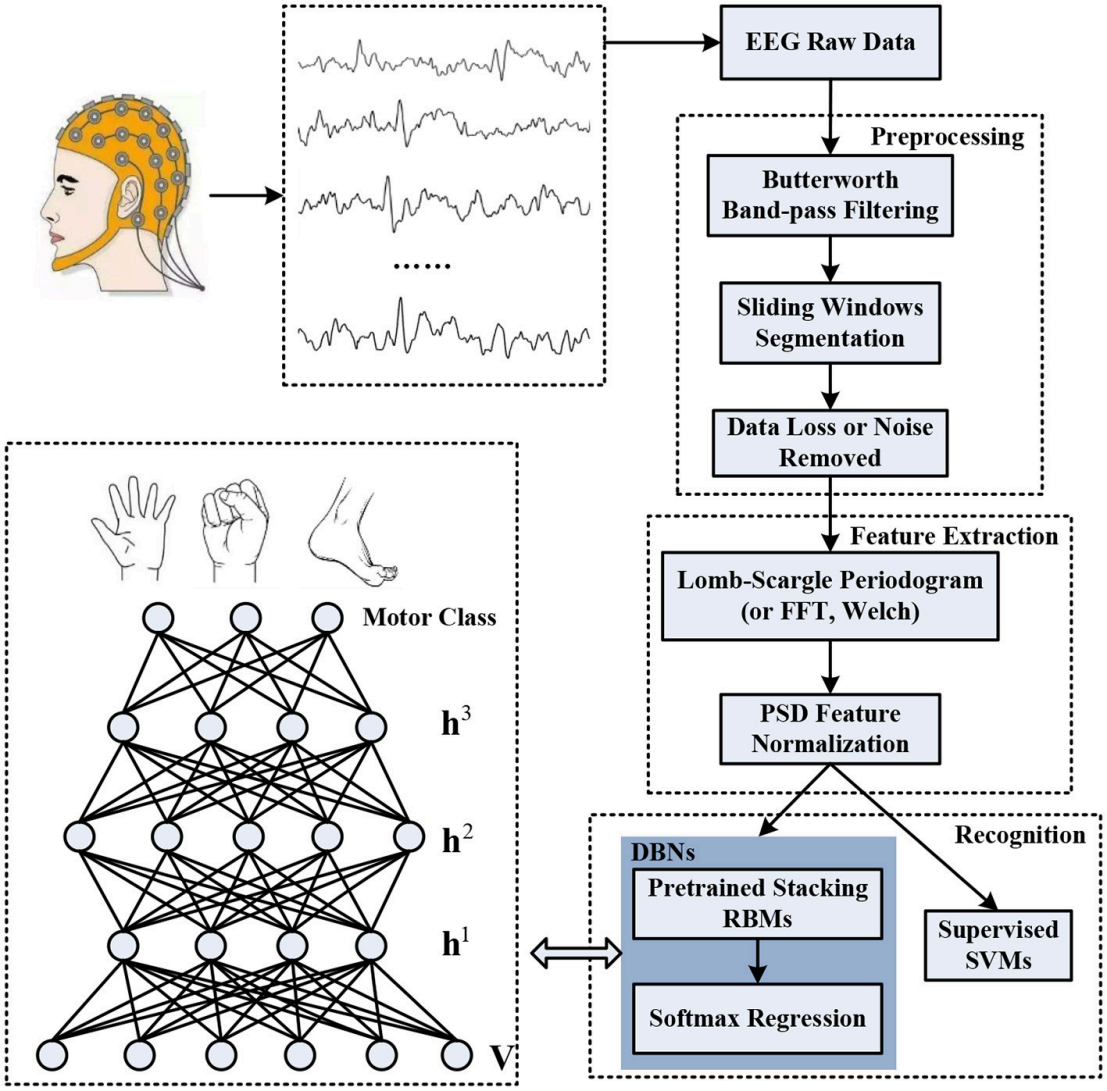
The overall decoding scheme for incomplete motor imagery EEG signals based on deep belief network (DBN).

The objective of our study is to address the issue of improvement of the recognition accuracy and stability associated with different motor imagery tasks for the incomplete EEG signals. The schematic diagram of the overall decoding system is illustrated in Figure 1, which primarily synergizes three procedures: preprocessing for raw EEG, spectral power feature extraction, and motor imagery recognition. Definitely, the raw EEG signals were captured by the means of non-invasive wet electrodes arranged on the brain scalp when individuals perform diverse motor imagery tasks, such as imagining limbs movements. The preprocess procedure was devoted to constructing incomplete motor imagery EEG datasets, which covered band-pass filtering, sliding windows segmentation, and data loss or noise removal. The deep belief network was composed of three layers of pre-trained stacking RBMs along with an output layer of softmax regression. The spectral power features within specific frequency bands extracted through Lomb-Scargle periodogram were normalized to pre-train each layer of the RBMs and fine-tune the weights of the DBN. Stochastic binary units were utilized in the pre-training stage to initialize the deep neural network. Deterministic real-valued probabilities were also implemented to adjust the connection weights of each layer by error backpropagation algorithm. After a fine-tuning stage, the trained DBN was employed to decode the corresponding classes of motor imagery from incomplete EEG, such as movement intention of left hand, right hand, or foot. The structure of each layer in the DBN was optimized and determined by various group experiments. Moreover, simulated and extensive experiments for multi-subjects, different feature extraction methods (FFT or Welch) and classifiers (supervised Support Vector Machines, SVMs) were conducted to verify the viability and effectiveness of the proposed decoding scheme for incomplete motor imagery EEG signals.

## EEG processing pipeline

### Preprocessing

In order to exclude the unwanted components of the interested EEG segments, the preprocessing procedure was designed to transform the intact EEG with complex artifacts or data loss into incomplete EEG segments. Essentially, the preprocessing pipeline consists of three sub-parts: (a) signal filtering, (b) sliding windows segmentation, and (c) artifacts or data loss removal. More explicitly, the signal filtering was dedicated to alleviating the background noises arose from experimental, instrumental, and electrical or physiological sources. The sliding windows were mainly responsible to segment the expected motor imagery fragments from the continuous EEG signals. For the motor imagery EEG segments, the portions with extreme artifacts or data loss were directly discarded and the remaining portions were utilized to form incomplete signals.

#### Signal filtering

Because of the fact that EEG signals contain useful information below 100 Hz, noise elements above this frequency may be directly excluded through low-pass filters. For motor imagery EEG, the phenomenon of ERS/ERD obviously appears in the frequency range of mu (8–12 Hz) and beta (18–26 Hz) rhythm band (Pfurtscheller et al., 2006). In other words, the frequency band of 8–30 Hz possesses the most discriminative information associated with different motor imagery tasks. In this study, a fifth-order Butterworth band-pass filter with gain 1.5, cutoff frequencies [8, 35] Hz was applied to attenuate the frequency component of specific noises while amplifying interested frequency band for motor imagery classification. After signal filtering, a large part of noise can be removed, such as EMG (high frequency noise, higher than 35 Hz), low frequency component of EOG (lower than 8 Hz) and electrical line interference (50 or 60 Hz). In addition, the baseline drift caused by head or limb motions can also be alleviated to reduce the impact on the raw EEG signals.

#### Sliding windows segmentation

For a continuous recorded EEG signal, we just only focus on the motor imagery segments. Then, the band-filtered and continuous EEG signals were segmented by a time window, which corresponding to a trial of motor imagery task. Moreover, a trial of motor imagery task needs repeatedly imagine limb movements for a certain time to generate stable and effective brain activity. In existing motor imagery EEG studies, the features can be extracted either by using the whole EEG trial or by dividing the trial into a number of overlapping/non-overlapping time segments (Asensio-Cubero et al., 2011, 2013; AYDEMIR, 2016). To improve the temporal resolution of EEG and obtain better performance of the classifier, a sliding window was commonly adopted to split the targeted motor imagery trial into overlapped segmentations which can be used for multiple classifications by a voting strategy (Herman et al., 2008; Shahid and Prasad, 2011; Choi, 2012). In this study, instead of using the whole data length of EEG trial, a four-second EEG trial was divided into 16 segments of 1 s length with 0.2 s step size by the 1 s sliding window with 80 % overlap.

#### Artifacts or data loss removal

Even if the filter processing is done, some artifacts may still exist in the EEG segments. Furthermore, the residual elements stem from artifacts may overlap the effective frequency band correlated with motor imagery EEG. For instance, the EOG artifacts resulted from eye blinks are usually presented in the frequency band of 0–10 Hz. The high frequency elements of the EOG overlapping with ERS/ERD bands cannot be readily excluded by band-pass filters. On the other hand, the filters are in general ineffective in the case of the signal with data loss. Instead of rejecting the entire motor imagery EEG segments, an additional preprocessing implementation was proposed to address artifacts and data loss. For the case of the EEG segment contaminated by extreme artifacts, the entire EEG segment was divided into data chunks with different widths. The width which represents the number of data points in each data chunk can be generated according to a normal distribution with a mean of 10 and a standard deviation of 2. A form of data chunk removal was applied to directly discard data chunks which contain severe artifacts. In addition, for the case of data loss within the EEG segment, a form of data point removal was employed to eliminate acquisition outliers. For the two forms of data removal, the EEG portions contaminated by extreme artifacts or data loss within an EEG segment were directly discarded by a proportion from 10% to 80% in this study. For example, for the case of 10% data chunk removal, 10% data chunks in a 1 s EEG segment were randomly discarded. For the case of 10% data point removal, 10% data points (100 points in this study) in a 1s EEG segment (1,000 points) were randomly discarded. Subsequently, the remaining EEG data chunks or data points were combined to construct the incomplete motor imagery EEG segments.

### Feature extraction based on Lomb-Scargle periodogram

The crucial step in a BCI system is feature extraction, which is used to find mental task-related information and most discriminative representations from the brain activities for subsequent classification. The quality of extracted features highly affects the performance of the following recognition process. For motor imagery EEG signals, we concentrated on the spectral analysis during certain frequency bands. The non-parametric fast Fourier transform (FFT) and Welch periodogram methods have been confirmed to effectively estimate the spectral power features for the intact motor imagery EEG, such as power spectral density (PSD) (Herman et al., 2008; Djemal et al., 2016). However, due to the incomplete motor imagery EEG signals belong to a kind of non-uniformly sampled sequence, these methods may not extract stable spectral features. In our research, the Lomb-Scargle periodogram was adopted to estimate the spectral power features for incomplete motor imagery EEG segments. An incomplete EEG segment is denoted by *X* ∈ *R*^*C*×*N*^, where *C* is the number of channels and *N* is the length of signal points. For each channel, the signal series were denoted by *eeg*(*t*_*i*_), where *i* = 1, 2, …, *N*.

#### Lomb-Scargle periodogram

For signal series *eeg*(*t*_*i*_), the spectral power at frequency ω_*f*_ should be estimated by solving the following fitting problem of sum of squared differences:

(1) $$\begin{array}{l} \underset{\begin{array}{c} \alpha \geq 0\\ \phi \in \left[0,2\pi \right] \end{array}}{\min}\sum_{i=1}^{N}{\left[eeg(t_{i})-\alpha cos(\omega_{f}t_{i}+\phi )\right]}^{2}. \end{array}$$

For simplicity, the dependence of α and ϕ about ω_*f*_ was replaced by using

(2) $$\begin{array}{l} a=\alpha \cos\left(\phi \right)\;and\;b=-\alpha \sin\left(\phi \right). \end{array}$$

The fitting problem can be reformatted by the term of a and *b*:

(3) $$\begin{array}{l} \underset{a,b}{\min}\overset{N}{\underset{i=1}{\sum }}{\left[eeg\left(t_{i}\right)-acos\left(\omega_{f}t_{i}\right)-b\sin\left(\omega_{f}t_{i}\right)\right]}^{2}. \end{array}$$

The optimal parameters in the minimizing Equation (3) can be obtained by solving

(4) $$\begin{array}{l} \left[\begin{array}{c} â\\ \hat{b} \end{array}\right]=R^{-1}r \end{array}$$

where

(5) $$\begin{array}{l} R=\overset{N}{\underset{i=1}{\sum }}\left[\begin{array}{c} \cos\left(\omega_{f}t_{i}\right)\\ \sin\left(\omega_{f}t_{i}\right) \end{array}\right]\left[\begin{array}{cc} \cos\left(\omega_{f}t_{i}\right) & \sin\left(\omega_{f}t_{i}\right) \end{array}\right] \end{array}$$

and

(6) $$\begin{array}{l} r=\overset{N}{\underset{i=1}{\sum }}\left[\begin{array}{c} \cos\left(\omega_{f}t_{i}\right)\\ \sin\left(\omega_{f}t_{i}\right) \end{array}\right]eeg\left(t_{i}\right). \end{array}$$

The power at specific frequency ω_*f*_ corresponding to optimal parameters â and $\hat{b}$, is given as follows:

(7) $$\begin{array}{l} \frac{1}{N}{\overset{N}{\underset{i=1}{\sum }}\left(\left[\begin{array}{c} â\hat{b} \end{array}\right]\left[\begin{array}{c} \cos\left(\omega_{f}t_{i}\right)\\ \sin\left(\omega_{f}t_{i}\right) \end{array}\right]\right)}^{2}\\ =\frac{1}{N}\left[\begin{array}{c} â\hat{b} \end{array}\right]R\left[\begin{array}{c} â\\ \hat{b} \end{array}\right]\\ =\frac{1}{N}r^{T}{\text{R}}^{-1}\text{r}. \end{array}$$

Accordingly, the powers for each channel signal at all frequency ω can be obtained by

(8) $$\begin{array}{l} P\left(\omega \right)=\frac{1}{N}r{\left(\omega \right)}^{T}R{\left(\omega \right)}^{-1}r\left(\omega \right). \end{array}$$

Similarly, the estimation step was repeatedly executed for all channels of the incomplete motor imagery EEG segments to extract the corresponding spectral features. Previous researches demonstrated that significant power oscillations in response to various motor imagery tasks mostly located in 8–30 Hz bands (Pfurtscheller et al., 2006; Shahid and Prasad, 2011). In this article, the concerned band was divided into four sub-bands with a bandwidth of 5 Hz, including alpha (8–13 Hz), sigma (13–18 Hz), low beta (18–23 Hz), and high beta (23–28 Hz) rhythms. For each channel, the PSD features of each sub-band were computed by averaging powers within the frequency range. Hence, all PSD features for EEG segments were concatenated by channel arrangement into a feature vector:

(9) $$\begin{array}{l} V=\left[p_{11},p_{12},p_{13},p_{14},p_{21},p_{22},p_{23},p_{24},\cdots \;,p_{C1},p_{C2},p_{C3},p_{C4}\right] \end{array}$$

where *C* is the number of channels.

#### Feature normalization

Generally, the original features can be directly fed into a neural network or an SVM classifier to recognize which motor imagery class the current EEG signal belongs to. However, the spectral feature variations caused by various channels or different motor imagery trials may affect the performance of classifiers. To eliminate the variation factor of feature scale and accelerate the convergence of learning algorithm, a min-max normalization step was utilized in feature vector set *V*. Refer to (10), the raw features were divided by the difference of maximum and minimum to scale all the values between 0 and 1.

(10) $$\begin{array}{l} F{\left(m\right)}_{norm}=\frac{V\left(m\right)-v_{min}\left(m\right)}{v_{max}\left(m\right)-v_{min}\left(m\right)} \end{array}$$

where, *v*_*max*_(*m*) = *max*{*V*(*m*)}, *v*_*min*_(*m*) = *min*{*V*(*m*)},*m* ∈ *R*^4 × *C*^.

### Deep belief network based on restricted Boltzmann machines

Considering the advantages of high-speed and parallel computation, a neural network classifier is more suitable and efficient for the online BCI application and the trained parameters can be directly used to distinguish new EEG signals. Currently, a variety of deep learning architectures based on neural networks have been constructed and applied in motor imagery EEG classification (Yang et al., 2015; Kumar et al., 2016; Tabar and Halici, 2016). In this study, we adopted a deep belief network (DBN) structure to obtain more robust and ultimately more notable representation for the incomplete motor imagery EEG. The DBN structure can be formed by multiple layers of stacked restricted Boltzmann machines (RBMs) or auto-encoders.

#### Restricted Boltzmann machine (RBM)

**Figure 2:**
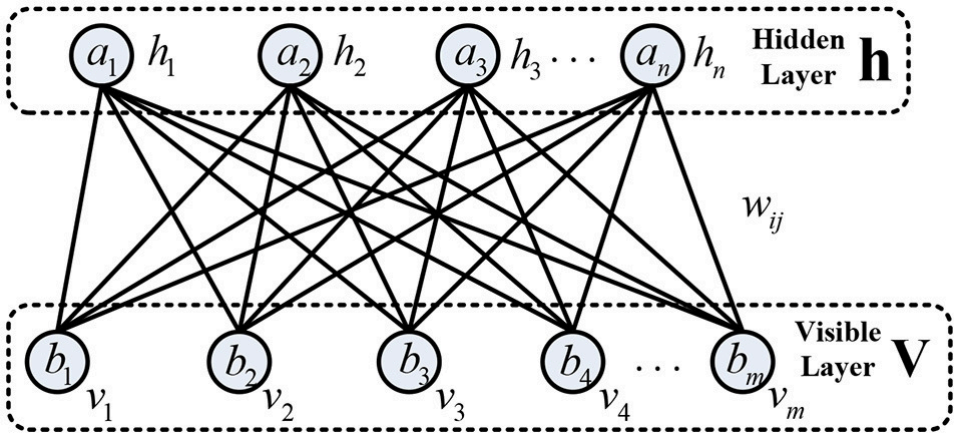
The basic structure of restricted Boltzmann machine (RBM).

Each RBM is composed of a visible layer, a hidden layer, and connection weights between two layers, which is greedily trained in an unsupervised mode (Hinton et al., 2006; Tang et al., 2015). The basic structure of RBM is presented in Figure 2. The neurons used in the RBM are stochastic binary units. Traditionally, the visible layer receives the input data and have undirected connections with the neurons of the hidden layer. Meanwhile, the neurons from the same layer are disconnected. The hidden layer is responsible to reconstruct the input data as close as possible by tuning the connection weights and biases repeatedly. For motor imagery EEG, each visible neuron represents a spectral feature with hypothetically Gaussian distribution. The energy function of joint configuration for the two layers is defined as

(11) $$\begin{array}{l} E\left(v,h\right)=-\overset{m}{\underset{i=1}{\sum }}b_{i}v_{i}-\overset{n}{\underset{j=1}{\sum }}a_{j}h_{j}-\overset{m}{\underset{i=1}{\sum }}\overset{n}{\underset{j=1}{\sum }}v_{i}h_{j}w_{ij} \end{array}$$

where *v*_*i*_ and *h*_*j*_ are the binary states at the visible neuron *i* and hidden neuron *j* respectively. *b*_*i*_ and *a*_*j*_ are the corresponding biases of neurons, *w*_*ij*_ is the connection weight between them. Based on the Boltzmann distribution and energy function, a joint probability for pair of the visible and hidden layer is determined by

(12) $$\begin{array}{l} p\left(\text{v},\text{h}\right)=\frac{1}{Z}e^{-E\left(\text{v},\text{h}\right)} \end{array}$$

where $Z=\underset{v,h}{\sum }e^{-E\left(v,h\right)}$ denotes the partition function or normalization term.

Considering that the hidden neurons are conditional independent due to no connections between them, given visible vector **v**, the conditional probability of neuron *h*_*j*_ being 1 can be obtained as follows:

(13) $$\begin{array}{l} p\left(h_{j}=1|\text{v}\right)=\sigma \left(a_{j}+\underset{i}{\sum }v_{i}w_{ij}\right) \end{array}$$

Similarly, given hidden vector *h*, the conditional probability of the visible neuron *v*_*i*_ being 1 can be determined by

(14) $$\begin{array}{l} p\left(v_{i}=1|\text{h}\right)=\sigma \left(b_{i}+\underset{j}{\sum }h_{j}w_{ij}\right) \end{array}$$

where σ(•) denotes the logistic sigmoid function.

Given the training dataset $S=\left\{s^{1},s^{2},\ldots ,s^{n_{s}}\right\}$, *n*_*s*_ is the number of training samples, the parameters of RBM are trained to fit the training samples by maximizing a log-likelihood function, including connection weights ***w***, biases ***a*** and ***b***.

(15) $$\begin{array}{l} L_{S}=\overset{n_{s}}{\underset{i=1}{\sum }}\log p\left(\text{v},\text{h}\right) \end{array}$$

Based on gradient ascent and contrastive divergence methods (Hinton et al., 2006), the derivative of the log-likelihood with respect to weights **w** can be formulized by

(16) $$\begin{array}{l} \frac{\partial \log p\left(\text{v},h\right)}{\partial w_{ij}}=E_{data}\left[\frac{\partial E\left(\text{v},\text{h}\right)}{\partial w_{ij}}\right]-E_{model}\left[\frac{\partial E\left(\text{v},\text{h}\right)}{\partial w_{ij}}\right] \end{array}$$

where ***E***_*data*_[•] and ***E***_*model*_[•] are respectively the expectation under the distribution of the training dataset and the model. Furtherly, the gradient can be rewritten by

(17) $$\begin{array}{l} \frac{\partial \log p\left(\text{v},\text{h}\right)}{\partial w_{ij}}=E_{data}\left[v_{i}h_{j}\right]-E_{model}\left[v_{i}h_{j}\right] \end{array}$$

The contrastive divergence method can be used to approximately estimate the expectation ***E***_*data*_[*v*_*i*_*h*_*i*_]. The Gibbs sampling method can be adopted to calculate the expectation ***E***_*model*_[*v*_*i*_*h*_*i*_]. Hence, the learning rule of connection weights can be obtained by

(18) $$\begin{array}{l} \Delta w_{ij}=\eta \left(E_{data}\left[v_{i}h_{i}\right]-E_{model}\left[v_{i}h_{i}\right]\right) \end{array}$$

Similarly, the updating rules of the biases are respectively

(19) $$\begin{array}{l} \Delta b_{i}=\varepsilon \left(E_{data}\left[v_{i}\right]-E_{model}\left[v_{i}\right]\right) \end{array}$$

and

(20) $$\begin{array}{l} \Delta a_{j}=\varepsilon \left(E_{data}\left[h_{j}\right]-E_{model}\left[h_{j}\right]\right) \end{array}$$

where η and ε donate the learning rate. According to the updating rules of parameters, each RBM is trained to reconstruct the input data in an unsupervised way.

#### Deep belief network

Three layers of RBM were superposed to construct a deep belief network with a layer of softmax regression in the study, as shown in Figure 1. The raw input data was fed to the bottom layer of RBM, and the output of the hidden layer from the lower RBM was delivered to the visible layer from the higher RBM. Compared to logistic regression, the softmax regression was used to solve multiclass recognition problems by statistically estimating the maximum probability of the class that a sample belongs to (Salakhutdinov and Hinton, 2012). The procedures of the DBN primarily consisted of pre-training stage and fine-tuning stage. The pre-training stage was conducted in each layer of RBM to obtain initial parameters of the DBN. The softmax regression was added to obtain prediction error to optimize the parameters by backpropagation algorithm in the fine-tuning stage. Additionally, some constraint terms were incorporated into the cost function of softmax regression to avoid overfitting, including weight decay and sparsity constraint (Cho, 2013; Plis et al., 2014; Jiang et al., 2016). In our research, the weight decay was set to 0.05 and the sparsity constraint was set to 0.1. The learning rates for connection weights and biases were set to 0.5 and 0.25 respectively. All these parameters were determined and optimized by a grid search procedure with 5-fold cross-validation.

## Motor imagery experimental paradigm and datasets

In our study, nine right-handed volunteers (all males, mean age 26.5 years, ranging from 25 to 28 years, numbered S01-S09) with thin hair participated in the motor imagery experiments. All subjects were healthy, without any history of neurological, psychiatric or cognitive disorders. Specifically, none of them has any prior experience of the BCI experiment related to motor imagery. Moreover, details of motor imagery experimental procedures were explained to all participants and written informed consents were signed for all subjects before the experiment. The experimental protocol was reviewed and approved by the local ethics committee of the University of Chinese Academy of Sciences.

**Figure 3:**
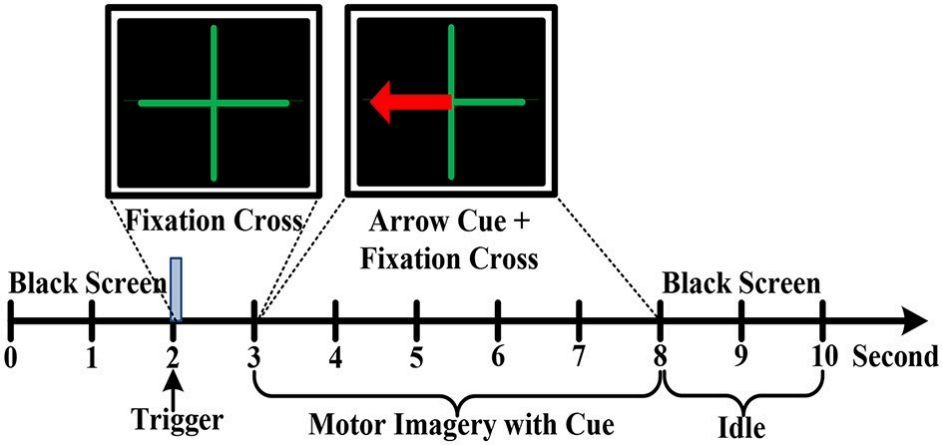
The motor imagery EEG experimental paradigm.

In an electromagnetic shielding environment, the participants were seated in a comfortable chair with armrests and watched an LCD screen from a distance of about 1 m, while wearing an EEG recording cap. Three kinds of motor imagery tasks were performed including imagining left hand, right hand and foot movements. Before the experiment, the instructor explained the meaning of kinesthetic imagery of the limb movements to the participants. Additionally, all participants performed motor imagery practice to get familiar with the kinesthetic sensation. Each participant carried out an experimental block consisted of 10 sessions, which lasted ~1.5 h. All sessions were executed in the same condition and a rest period with several minutes was given between two consecutive sessions. The experiment paradigm of each session was devised in Figure 3. For all sessions, the first 2 s was an idle state with a black screen. Subsequently, a fixation green cross was emerged at the center of the screen with a duration of 1 s to indicate the beginning of one trial. Immediately, a red arrow pointing to the left, right or down appeared with a duration of 5 s in addition to the fixation cross. In this specific period, the subjects were instructed to respectively perform the relevant motor imagery tasks according to the direction of the arrow, such as imagining repeated finger flexion and extension with the left or right hand at approximate 1 Hz frequency. Meanwhile, the subject must pay attention to imagine the kinesthetic experience of limb movements as much as possible. In addition, to minimize the artifacts, the participants were asked to limit their head movements and try not to blink or swallow during the motor imagery period. During the inter-trial interval, the arrow cue and fixation cross were disappeared with the remaining of a black screen for 2 s, and the subject was instructed to perform idle state instead of motor imagery. To avoid the adaptability of brain activity for a given motor imagery task, each of the 3 cues was presented 10 times by a random sequence in each session. Hence, there are 30 trials for a session. For each subject, there are total 300 trials of motor imagery tasks in an experiment.

During the motor imagery tasks, EEG signals were collected through a grid cap with 64 Ag/AgCI passive electrodes provided by Plexon Inc., USA. The multiple electrodes with roughly 3 cm separation distance were closely arranged on the cap according to the international 10–20 positioning system. Extra conductive glues or gels were injected into each electrode for higher conductivity and better attachment. The left mastoid electrode was used as the reference channel and the right mastoid electrode served as the ground. The original EEG data were recorded with a sampling rate of 1 kHz by OmmiPlex Neural Data Acquisition System (Plexon Inc., USA), including analog pre-amplification, analog-to-digital conversion, and a low-pass filter with a cutoff frequency of ~200–300 Hz. An additional notch filter with 50 Hz was applied to eliminate the power line artifacts. Finally, the recorded motor imagery EEG signals for each subject were saved in the form of times × channels × trials with 5,000 × 64 × 300.

To obtain dominant motor imagery EEG, a 4 s segment from 0.5 s after cue to 4.5 s was cut out from each trial. As mentioned in section EEG Processing Pipeline, the data was further band-pass filtered and segmented by a sliding window. Hence, the motor imagery datasets were represented by a three-dimensional array of size 1,000 × 64 × 4,800 for each subject, where 1000 was the length of time window (1 s), 4,800 was the number of motor imagery segments containing three class, and 64 was the number of channels. For each channel signal, there were 4 spectral power features estimated by Lomb-Scargle periodogram method. Then, the whole sample datasets with features were 4,800 × 256 for each subject, where 256 was the number of features (4 × 64 channels). The datasets were randomly divided into 75% training datasets (3,600 × 256) and 25% testing datasets (1,200 × 256).

## Experimental results and discussions

### Simulation comparison with different spectral estimation methods

To evaluate the effectiveness of the Lomb-Scargle method for incomplete signals, the simulated signal was devised by mixing two sinusoidal signals with a dominant frequency of 4 Hz and 8 Hz, respectively. The amplitude ratio between 4 Hz and 8 Hz sinusoidal signal was set to 0.75. For the simulated signal, data points with a certain proportion were randomly removed to construct incomplete or irregular signals. In addition, for comparison with Lomb-Scargle periodogram, traditional Welch and FFT periodogram methods were also applied to estimate spectral power for different incomplete signals.

**Figure 4:**
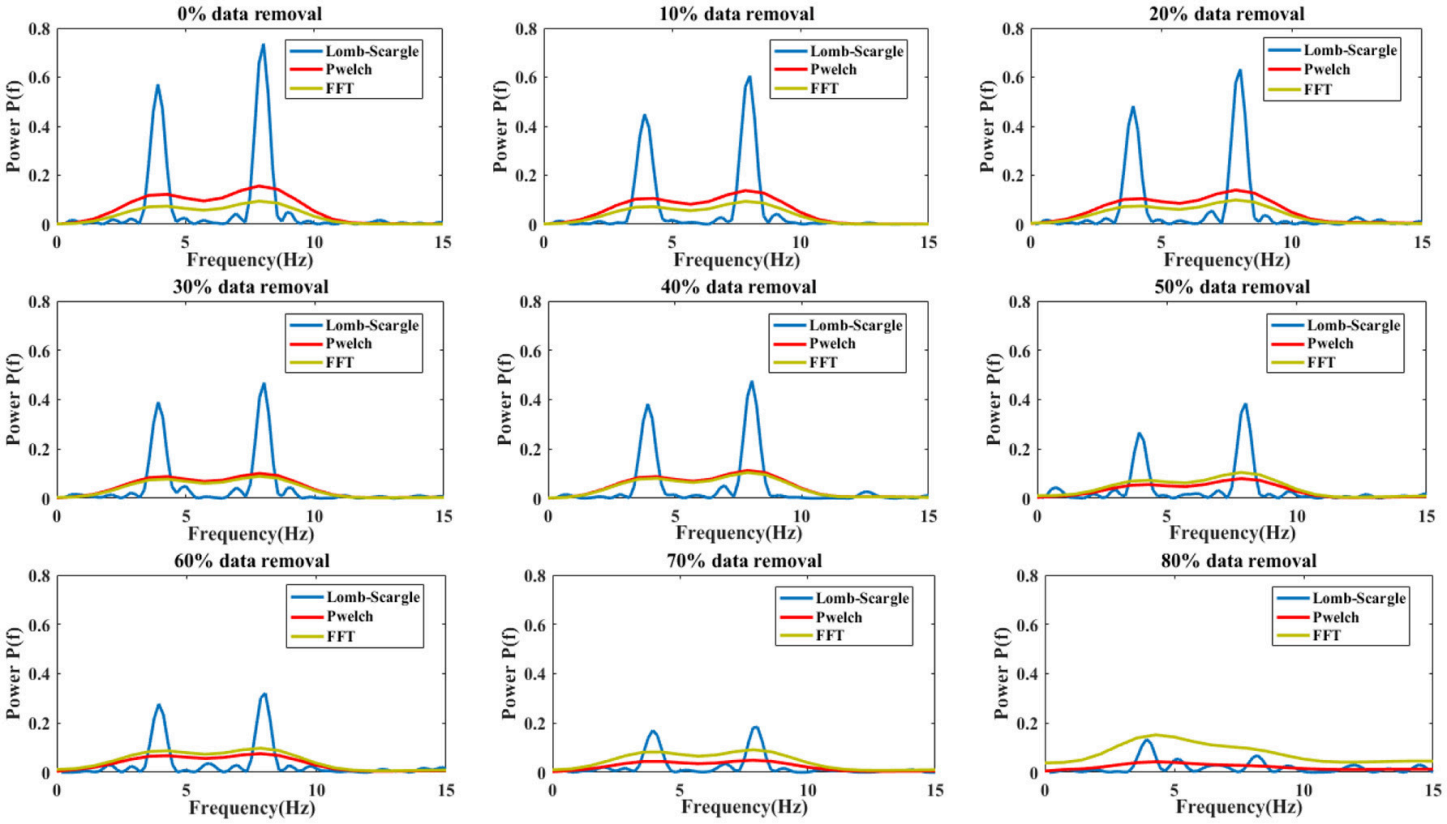
The comparison results of spectral power estimations for the complete signal and incomplete signal with different proportional removal (from 10 to 80% with a step of 10%). Three estimation methods were used: Lomb-Scargle, Welch and FFT periodogram.

The estimated spectral powers for the intact signal and the incomplete signal with various degrees of missing data are given in Figure 4. For the simulated signal, the data points were eliminated by a proportion from 10 to 80% with a step of 10%. Meanwhile, the powers were normalized to the same scale by dividing a factor, which was the proportion value of remaining data. From Figure 4, we can see that the spectral components at dominant frequency 4 and 8 Hz are more and more insignificant with the increase of proportional data removal for all three estimation methods. Especially, the spectral powers were obviously degraded after 30% data removed. However, the spectral powers estimated by Lomb-Scargle periodogram were more notable than those estimated by Welch or FFT method for various incomplete signals (the *p*-value from paired *t*-test was < 0.05). Indeed, the components at 4 Hz and 8 Hz were well-obtained for the incomplete signal even with 80% data removed. It demonstrated that compared to the traditional spectral analysis methods like FFT and Welch, the LSP method can estimate more stable and optimal spectral features from various incomplete or irregular signals. It proved that the LSP was particularly suited to estimate rhythm components in non-uniformly sampled signals (Stoica et al., 2009).

### Incomplete motor imagery EEG: point removal form and chunk removal form

**Figure 5:**
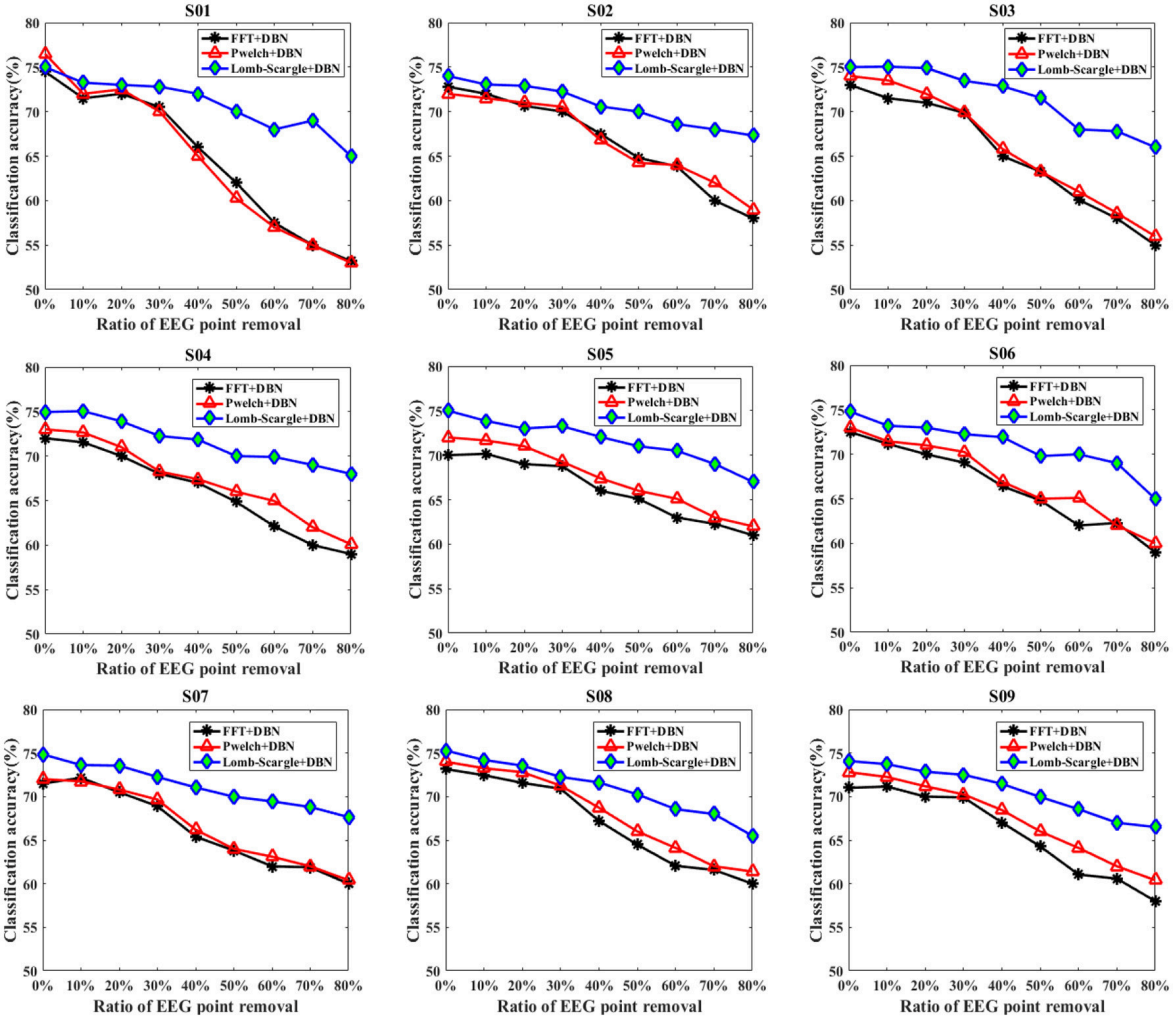
The classification results of the intact EEG and incomplete EEG with various ratios of data point removal (from 10 to 80% with a step of 10%), for the nine subjects (from S01 to S09). Three spectral feature extraction methods were used for comparison: the black lines, red lines and blue lines represent the accuracy of DBN with FFT, Welch and Lomb-Scargle feature extraction, respectively.

To systematically validate the discrimination ability of the PSD features extracted by the LSP method for the incomplete EEG, two forms were adopted to randomly remove the portions from the intact motor imagery segments to construct incomplete signals. For the condition of data loss, a form of data point removal was applied to eliminate the EEG outliers, which caused by high contact impedance between electrodes and scalp. Figure 5 presents the recognition performance of intact EEG and incomplete EEG with different proportions of data point removal for the nine subjects, obtained by the DBN classifier with three feature extraction methods (FFT, Welch, and Lomb-scargle). For simplify, three methods were denoted as FFT+DBN, Welch+DBN, and Lomb-Scargle+DBN, respectively. From an overall perspective, the recognition accuracy showed a descending trend gradually along with the increasing proportion of data point removal for all three methods in Figure 5. For the intact motor imagery EEG, the average accuracies (±standard deviation) across the nine subjects were 72.27% (±1.33%) for FFT+DBN, 73.26% (±1.44%) for Welch+DBN, 74.77% (±0.43%) for Lomb-Scargle+DBN, respectively. There was no significant difference (*p* > 0.078, paired *t*-test) between the average accuracy of Lomb-Scargle+DBN and those of the other methods for the intact EEG across all subjects. This can be inferred that compared to the FFT and Welch method, the LSP method may not provide high-quality PSD features for the intact motor imagery EEG. Especially, for the intact EEG of subject 1 (S01), the accuracy of Welch+DBN was higher than that of Lomb-Scargle+DBN. Considering the computational complexity and the efficiency, it is not preferable to apply the Lomb-Scargle+DBN for the intact motor imagery EEG classification. However, the accuracy variation of Lomb-Scargle+DBN was obviously smaller than those of the FFT+DBN and Welch+DBN for the incomplete EEG with different point removal ratios. More specifically, for the incomplete EEG with point removal in the range from 10 to 80%, the mean difference of accuracy across the nine subjects was 13.38% (±2.67%) for FFT+DBN, 13.08% (±3.07%) for Welch+DBN, and 7.45% (±1.18%) for Lomb-Scargle+DBN, respectively. It demonstrated that the classification performance of Lomb-Scargle+DBN was significantly better compared to FFT+DBN (*p* = 0.012 < 0.05, paired Student's *t*-test) and Welch+DBN (*p* = 0.008 < 0.01, paired Student's *t*-test) for the incomplete motor imagery EEG. Implicitly, the spectral power features extracted by Lomb-Scargle periodogram can significantly improve the classification accuracy of the DBN for various degrees of incomplete EEG. An acceptable classification accuracy (above 65%) can be achieved by the Lomb-Scargle+DBN method even when 80% of points were eliminated, while the accuracies of FFT+DBN and Welch+DBN were ~60% or even lower. Interestingly, from Figure 5, we can find that the accuracies for the incomplete EEG after 30% data point removal declined sharply and substantially. Especially in the case of subject 1 (S01 EEG datasets), the accuracy obtained by FFT+DBN or Welch+DBN roughly varied from 70 to 53% for the incomplete EEG between 30 and 80% data point removal. This finding implied that the performance of spectral power features deteriorated distinctly for the methods of FFT and Welch periodogram, which was in accordance with the previous simulation comparison.

**Figure 6:**
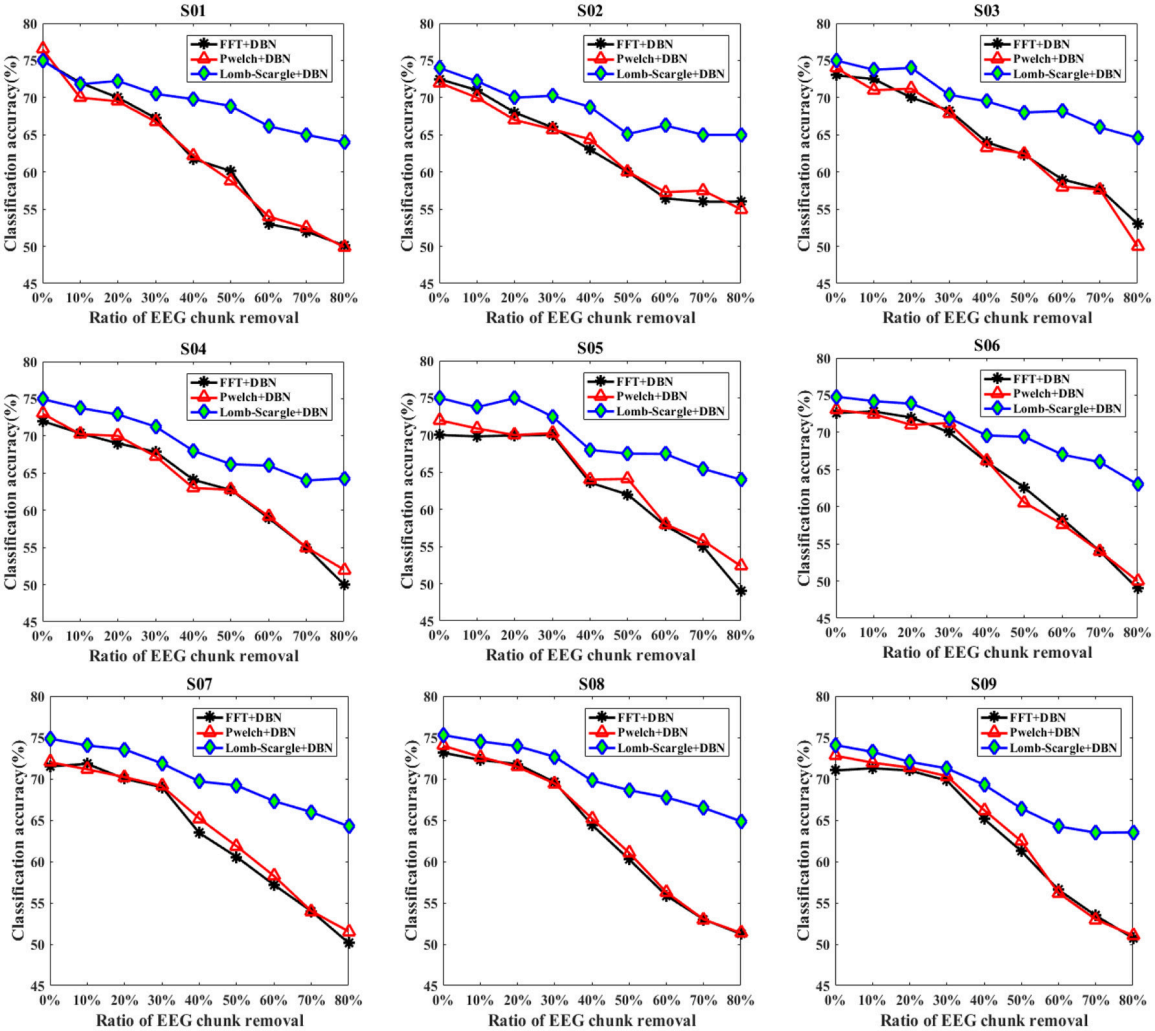
The classification results of intact EEG and incomplete EEG with various ratios of data chunk removal (from 10 to 80% with a step of 10%), for the nine subjects (from S01 to S09). Three spectral feature extraction methods were used for comparison: the black lines, red lines and blue lines represent the accuracy of DBN with FFT, Welch and Lomb-Scargle feature extraction, respectively.

Similarly, to eliminate the effects of extreme artifacts, a form of data chunk was adopted to remove the EEG portions contaminated by tremendous electrophysiological artifacts or complex background noises. The corresponding classification results for the intact EEG and incomplete EEG with various ratios of data chunk removal are presented in Figure 6. Compared to the data point removal, the accuracies of the incomplete EEG dramatically and significantly decreased across different degrees of data chunk removal (*p* = 0.022 < 0.05, paired Student's *t*-test). Especially, the average accuracies for the incomplete EEG with 80% data chunk removal were 51.03% (±2.23%), 51.47% (±1.60%), and 64.17% (±0.63%), significantly lower than those for the incomplete EEG with 80% data point removal by 58.13% (±2.52%), 59.15% (±2.87%), and 66.44% (±1.13%) for FFT+DBN, Welch+DBN, and Lomb-Scargle+DBN respectively. More commonly and exactly, the mean difference of accuracy for the incomplete EEG with chunk removal in the range from 10 to 80% across the nine subjects was 20.51% (±2.39%), 19.68% (±2.21%), and 9.30% (±1.17%) for FFT+DBN, Welch+DBN, and Lomb-Scargle+DBN respectively. The statistical analysis indicated that the proposed Lomb-Scargle+DBN method for the incomplete EEG was constantly and significantly superior to the other two methods (*p* = 0.007 < 0.01 for FFT+DBN and Lomb-Scargle+DBN, *p* = 0.007 < 0.01 for Welch+DBN and Lomb-Scargle+DBN, paired Student's *t*-test). Moreover, the accuracies of the incomplete EEG in the condition of data chunk removal varied remarkably larger than those in the condition of data point removal (*p* < 0.05, paired *t*-test). It can be attributed to the fact that except for extreme artifacts, the informative signals corresponding to motor imagery tasks were also eliminated by the chunk form within the same contaminated segments. Thereby, for the incomplete EEG with data chunk removal, the extracted spectral powers of the mu/beta rhythms related to motor imagery tasks were relatively inferior to those for the incomplete EEG with data point removal.

In addition, the overall recognition performance for the incomplete EEG across various degrees of point and chunk removal are provided in Table 1. The results (mean ± standard deviation) were obtained by averaging accuracies for the incomplete EEG with different ratios of point and chunk removal in the range from 10 to 80%. It can be observed that the classification results of Lomb-Scargle+DBN were significantly higher than those of FFT+DBN and Welch+DBN for both incomplete EEG with point and chunk removal. The incremental performances between Lomb-Scargle+DBN and FFT+DBN were 5.48%, 6.60% for the incomplete EEG with point and chunk removal, respectively. The *p*-values computed by the paired Student's *t*-test of this comparison were all < 0.001. Likewise, the incremental performances between Lomb-Scargle+DBN and Welch+DBN were 4.67%, 6.44% for the incomplete EEG with point and chunk removal, respectively. The *p*-values computed by the paired Student's *t*-test of this comparison were also < 0.001. Furthermore, from the view of standard deviation, the Lom-Sacrgle+DBN method (2.68% for point form, 3.58% for chunk form) performed prominently lower variability than FFT+DBN (5.08% for point form, 7.70% for chunk form) and Welch+DBN (4.93% for point form, 7.49% for chunk form). Therefore, it is evident that the Lomb-Scargle+DBN method can significantly and steadily improve the recognition performance for the different incomplete motor imagery EEG.

**Table 1:** Statistical classification performance for the incomplete EEG with point and chunk removal.

	**Incomplete EEG (point form) (%)**			**Incomplete EEG (chunk form) (%)**		
	**FFT**	**Welch**	**Lomb-Scargle**	**FFT**	**Welch**	**Lomb-Scargle**
S01	63.46 ± 7.64	63.09 ± 7.85	70.38 ± 2.93	60.78 ± 8.50	60.46 ± 7.88	68.54 ± 3.13
S02	65.85 ± 5.10	66.14 ± 4.60	70.34 ± 2.25	62.06 ± 5.86	62.12 ± 5.40	67.82 ± 2.84
S03	64.21 ± 6.24	65.01 ± 6.40	71.20 ± 3.49	63.35 ± 6.67	62.69 ± 7.34	69.30 ± 3.36
S04	65.31 ± 4.62	66.54 ± 4.24	71.24 ± 2.44	62.25 ± 7.17	62.43 ± 6.72	68.30 ± 3.85
S05	65.66 ± 3.41	66.93 ± 3.55	71.21 ± 2.34	62.15 ± 7.80	63.18 ± 7.12	69.20 ± 4.02
S06	65.59 ± 4.32	66.46 ± 4.24	70.52 ± 2.72	63.09 ± 8.73	62.89 ± 8.59	69.36 ± 3.92
S07	65.58 ± 4.46	65.99 ± 4.30	70.79 ± 2.21	62.01 ± 8.46	62.66 ±7.52	69.48 ± 3.53
S08	66.28 ± 4.94	67.44 ± 4.75	70.79 ± 2.99	62.31 ± 8.46	62.55 ± 8.39	69.83 ± 3.56
S09	65.25 ± 5.00	66.83 ± 4.40	70.34 ± 2.75	62.40 ± 8.16	62.82 ± 8.48	67.94 ± 4.02
Mean	65.24 ± 5.08	66.05 ± 4.93	**70.72** ± 2.68	62.26 ± 7.70	62.42 ± 7.49	**68.86** ± 3.58

*The maximum mean of comparative experiments were highlighted in the bold*.

### Comparison of DBN with various structures

It should be noted that the structures of DBN adopted in the incomplete EEG experiments were determined and selected by an optimization method. As previously mentioned, the DBN was constructed by three hidden layers of pretrained RBMs and an output layer of softmax regression. For this study, a number of 256 dimensional vectors were fed to the input layer of the DBN. Hence, the dimension of the input layer was 256. Furthermore, three units were utilized in the output layer of softmax regression, which corresponded to three motor imagery tasks. To obtain the relevant optimal parameters, various numbers of units were tried for the three hidden layers. More explicitly, different numbers of units varied over a range were used in one hidden layer, while the numbers of units in the remaining two hidden layers were unchanged. Since optimal parameters selection of the DBN was a combinatorial process, which yields comparable solutions rapidly. To evaluate the sensitivity of the hidden layers to the changes of the unit numbers, 5-fold cross-validation was applied for the classification of motor imagery EEG. For each subject, the intact EEG and incomplete EEG with various ratios of data removal were divided into 5 sections, in which 4 sections were adopted for training, and the rest section was used for the test. The average performances were obtained by executing 5 times procedures repeatedly. Additionally, all the evaluations were conducted in the features extracted by the Lomb-Scargle periodogram.

For the first hidden layer, the numbers of units varied in a range of [15 30 45 60 75 90] while the numbers of units in the other two hidden layers maintained a constant value with 50 and 35 units, respectively. The corresponding comparison of classification performances for the DBN with different numbers of units in the first hidden layer is presented in Table 2. The results showed that the maximum mean accuracy 71% was obtained in the condition of 60 units of the first hidden layer. The decoding accuracies were remarkably improved in the 60 units compared to other numbers of units for the first hidden layer (*p* < 0.05, paired Student's *t*-test). Similarly, Table 3 gives the performance of the second hidden layer varying in [10 20 30 40 50 60] units with the other two hidden layers of 60 and 35 units respectively. The accuracies of 50 units in the second hidden layer (about 72%) were significantly higher than those of other numbers of units (*p* < 0.05, paired Student's *t*-test). Table 4 represents the results of the third hidden layer taking units from [25 30 35 50 70 85] when the other two hidden layers of 60 and 50 units respectively. It can be observed that the performances of 35 units in the third hidden layer were significantly different compared to the other numbers of units (*p* < 0.01, paired Student's *t*-test). The process of adjusting parameters was very tedious and tricky for the BDN. Nevertheless, the change of the classification accuracy was lower than 10% for the motor imagery tasks with different numbers of units in the three hidden layers. It suggested that the DBN classifier was robust relative to the variation of the network structure. In brief, the structure of the DBN used in this experiment was 256 × 60 × 50 × 35 × 3.

**Table 2:** Comparison of classification accuracies based on different numbers of units in the first hidden layer for the nine subjects.

	**15 units**	**30 units**	**45 units**	**60 units**	**75 units**	**90 units**
S01	0.62	0.65	0.68	0.68	0.60	0.63
S02	0.71	0.70	0.65	0.70	0.62	0.59
S03	0.60	0.58	0.62	0.73	0.60	0.58
S04	0.59	0.67	0.60	0.72	0.70	0.64
S05	0.61	0.63	0.64	0.71	0.62	0.59
S06	0.64	0.65	0.63	0.71	0.61	0.60
S07	0.63	0.65	0.67	0.69	0.64	0.63
S08	0.63	0.66	0.62	0.70	0.62	0.58
S09	0.62	0.64	0.62	0.71	0.62	0.63
Mean	0.63	0.65	0.64	**0.71**	0.63	0.61

*The maximum mean of comparative experiments were highlighted in the bold*.

**Table 3:** Comparison of classification accuracies based on different numbers of units in the second hidden layer for the nine subjects.

	**10 units**	**20 units**	**30 units**	**40 units**	**50 units**	**60 units**
S01	0.63	0.68	0.65	0.70	0.73	0.67
S02	0.63	0.67	0.65	0.68	0.72	0.66
S03	0.60	0.67	0.67	0.70	0.70	0.62
S04	0.62	0.70	0.70	0.69	0.73	0.67
S05	0.61	0.68	0.66	0.69	0.69	0.65
S06	0.62	0.67	0.69	0.68	0.74	0.67
S07	0.64	0.65	0.66	0.60	0.74	0.68
S08	0.60	0.62	0.70	0.70	0.75	0.65
S09	0.59	0.60	0.62	0.68	0.68	0.64
Mean	0.62	0.66	0.67	0.68	**0.72**	0.66

*The maximum mean of comparative experiments were highlighted in the bold*.

**Table 4:** Comparison of classification accuracies based on different numbers of units in the third hidden layer for the nine subjects.

	**25 units**	**30 units**	**35 units**	**50 units**	**70 units**	**85 units**
S01	0.60	0.62	0.72	0.66	0.65	0.70
S02	0.65	0.62	0.69	0.60	0.65	0.66
S03	0.64	0.68	0.70	0.70	0.66	0.65
S04	0.62	0.64	0.70	0.62	0.60	0.62
S05	0.62	0.63	0.71	0.62	0.64	0.63
S06	0.63	0.65	0.68	0.64	0.64	0.62
S07	0.60	0.66	0.68	0.63	0.68	0.64
S08	0.64	0.60	0.71	0.63	0.60	0.65
S09	0.61	0.60	0.70	0.65	0.62	0.65
Mean	0.62	0.63	**0.70**	0.64	0.64	0.65

*The maximum mean of comparative experiments were highlighted in the bold*.

### Comparison between DBN and SVM

In this series of experiments, performance comparisons between DBN and SVM were evaluated, with respect to the recognition accuracy for the incomplete EEG in the case of point removal and chunk removal respectively. As previously described, the Lomb-Scargle periodogram can extract effective and robust spectral features for various incomplete EEG to promote the classification performance. Hence, the DBN and SVM classifiers were executed on the same feature datasets extracted by the Lomb-Scargle method. For the three motor imagery tasks, three binary SVMs with a Radial Basis Function (RBF) kernel were built to obtain the final accuracy by a majority voting strategy. The relevant parameters of the binary SVM were optimized using a grid-search trick (Quitadamo et al., 2017) in a range of [−5 5], such as regularization parameter *C* and kernel width σ of the RBF. In addition, 5-fold cross-validation method was also applied to avoid overfitting for both classifiers.

**Figure 7:**
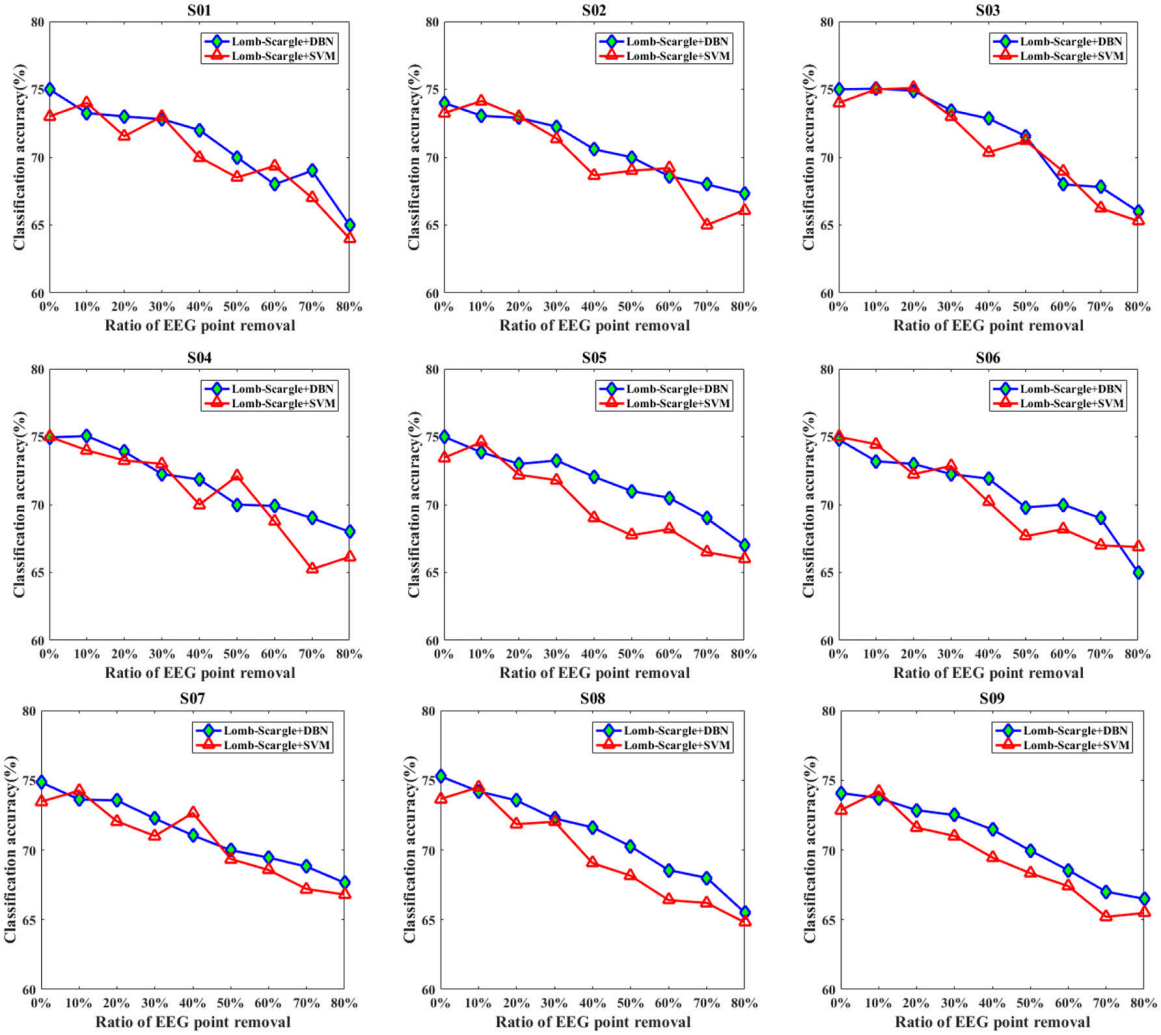
The comparative performances between DBN and SVM classifiers for the intact EEG and incomplete EEG with various ratios of data point removal (from 10 to 80% with a step of 10%), for the nine subjects (from S01 to S09).

**Figure 8:**
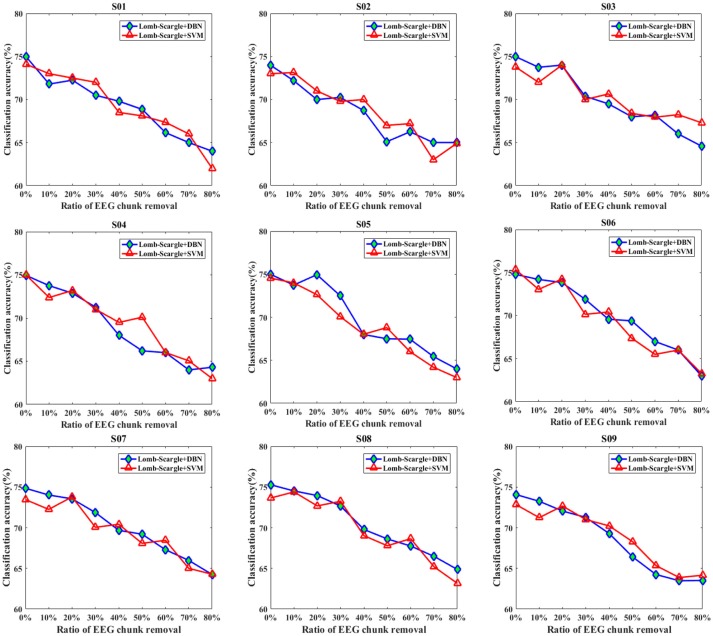
The comparative performances between DBN and SVM classifiers for the intact EEG and incomplete EEG with various ratios of data chunk removal (from 10 to 80% with a step of 10%), for the nine subjects (from S01 to S09).

Figures 7, 8 present the comparison results between DBN and SVM for the intact EEG and incomplete EEG in the case of point removal and chunk removal (ratios from 10 to 80% with a step of 10%), respectively. For the intact motor imagery EEG, the performance between DBN and SVM across the nine subjects was no significantly difference (*p* = 0.062 > 0.05, paired Student's *t*-test), with mean accuracies of 74.77% (±0.44%), 73.74% (±0.78%) respectively. From Figure 7, the overall performance of the DBN for the incomplete EEG with different ratios of point removal was better than that of the SVM. Especially, for the case of subject 5, 8, and 9 (S05, S08, and S09 EEG datasets), the accuracies of the DBN for the incomplete EEG after 30% data point removal were obviously improved, with an average increment of 2.64%. However, for the incomplete EEG with different ratios of data chunk removal, the accuracy improvement of the DBN was not significant compared with the SVM. For some subjects, such as subject 2, 3, 4, and 9, the SVM can outperform the DBN for the incomplete EEG with chunk removal in some degree (seen in Figure 8).

For further clarification, the average accuracies (± standard deviation) of the DBN and SVM across the incomplete EEG with various ratios of data removal (from 10 to 80% with a step of 10%) were presented in Table 5, including the case of point removal and chunk removal respectively. As shown, for the incomplete EEG with point removal method, the average classification performance of the DBN (70.72 ± 2.65%) was higher than that of the SVM (69.89 ± 3.08%) across the nine subjects. For the case of point removal, the *p*-value computing from the Student's *t*-test between DBN and SVM was 0.021 < 0.05. Moreover, the DBN led to relatively lower variability compared to the SVM, with a mean standard deviation of 2.65% and 3.08% respectively. These results indicated that the DBN was superior to the SVM for the incomplete EEG classification in terms of point removal. Whereas, in the case of chunk removal, the increase of accuracy between DBN (68.86 ± 3.58%) and SVM (68.74 ± 3.53%) was lower than that in the case of point removal. And there was no statistical difference between DBN and SVM (*p* = 0.79 > 0.50, paired Student's *t*-test) for the incomplete EEG with chunk removal. This may be due to the reason that compared to the incomplete EEG with point removal, the extracted features from the incomplete EEG with chunk removal were relatively poor and weaken the performance of the DBN and SVM. However, it is likely that the DBN can perform better than the SVM for the motor imagery classification of the incomplete EEG when parameters are subtly tuned and extra layers are added.

**Table 5:** Statistical classification performance of the DBN and SVM for the incomplete EEG with point and chunk removal.

	**Incomplete EEG (point form) (%)**		**Incomplete EEG (chunk form) (%)**	
	**DBN**	**SVM**	**DBN**	**SVM**
S01	70.38 ± 2.93	69.68 ± 3.26	68.54 ± 3.13	68.68 ± 3.75
S02	70.34 ± 2.25	69.56 ± 3.17	67.82 ± 2.84	68.25 ± 3.35
S03	71.20 ± 3.49	70.64 ± 3.70	69.30 ± 3.36	69.82 ± 2.31
S04	71.24 ± 2.44	71.24 ± 2.44	68.30 ± 3.85	68.20 ± 3.90
S05	71.21 ± 2.34	69.51 ± 3.04	69.20 ± 4.02	68.34 ± 3.87
S06	70.52 ± 2.72	69.93 ± 2.94	69.36 ± 3.92	68.75 ± 3.84
S07	70.79 ± 2.21	70.23 ± 2.68	69.48 ± 3.53	69.04 ± 3.30
S08	70.49 ± 2.99	69.12 ± 3.39	69.83 ± 3.56	69.27 ± 3.97
S09	70.32 ± 2.45	69.08 ± 3.11	67.94 ± 4.02	68.35 ± 3.47
Mean	**70.72** ± 2.65	69.89 ± 3.08	**68.86** ± 3.58	68.74 ± 3.53

*The maximum mean of comparative experiments were highlighted in the bold*.

## Conclusions and future works

In this study, a decoding scheme based on the combination of LSP and DBN was proposed to recognize incomplete motor imagery EEG segments. To construct incomplete EEG segments, point and chunk removal form were respectively utilized to randomly and proportionally eliminate the uninteresting EEG point or portion. The point removal form was mainly used to eliminate outliers within the EEG segments due to data loss. And the chunk removal form was used to eliminate portions within the EEG segments due to extreme artifacts. The LSP method was carried out to extract robust spectral power features of mu/beta rhythms related to motor imagery tasks for the incomplete EEG. The DBN consisted of three layers of stacking restricted Boltzmann machines (RBMs) and a softmax regression layer was devised to perform motor imagery classification. Since this was a preliminary study, the chunk and point removal was processed in a random manner. However, for the real application, a more specific search process was needed to determine which chunks or points should be removed.

To validate the effectiveness of the proposed decoding scheme for the incomplete EEG, various comparative experiments were conducted and evaluated on simulated signal and real motor imagery EEG, including the comparison of different spectral power estimation methods (FFT, Welch and Lomb-Scargle) and different classifiers (DBN and SVM). For the simulation comparison with three spectral estimation methods, the results show that the Lomb-Scargle method can extract more stable and remarkable spectral power for the incomplete or irregular signals. Furthermore, the PSD features extracted by the three estimation methods were recognized using a DBN classifier, and the classification accuracy of the Lomb-Scargle+DBN was not dramatically declined compared to FFT+DBN and Welch+DBN for the incomplete motor imagery EEG with increasing proportion of point removal or chunk removal (from 10% to 80% with a step of 10%). These results suggest that the Lomb-Scargle+DBN can lead to significantly and steadily improve the recognition performance for the incomplete motor imagery EEG. The significance statistical analysis between Lomb-Scargle+DBN and FFT+DBN or Welch+DBN was less than 0.05 for the incomplete EEG in the case of point removal and chunk removal. After three groups of experimental tests and comparisons, the structure of the DBN was determined to be 256 × 60 × 50 × 35 × 3 to improve the learning performance of the DBN. Extended comparison between DBN and SVM indicated that the DBN was superior to the SVM for the incomplete EEG in terms of point removal. Moreover, for the classification of the intact motor imagery EEG, there was no significant difference for the average accuracy (*p* > 0.078, paired *t*-test) between the Lomb-Scargle+DBN and the other methods (FFT+DBN and Welch+DBN). Considering the computational complexity and the efficiency, it is not preferable to apply the Lomb-Scargle+DBN for the intact motor imagery EEG classification. Therefore, the proposed decoding scheme is suitable to improve the classification performance for the incomplete motor imagery EEG. It means that instead of rejecting the entire segment, the motor imagery EEG segment with data loss or extreme artifacts can still be used to generate comparable classification results when the affected portions are eliminated.

Thanks to decoding the incomplete EEG, the proposed scheme will be beneficial to improve the stability, smoothness and maintain continuous outputs for a BCI system. Especially, for online BCI systems, the intentions of subjects are continuously decoded from the EEG signals with no interruption. In the future work, the online test based on motor imagery EEG will be carried out to evaluate the validity of the proposed decoding scheme for the incomplete signals. Additionally, because of the Lomb-Scargle periodogram was particularly suited to estimate rhythm components in non-uniformly sampled signals (Stoica et al., 2009), it may be applicable to other modalities of the EEG signal related to spectral analysis. For example, the proposed method can be applied to decode the incomplete SSVEP EEG. For the structure of the DBN, more dedicated procedures can be implemented to further boost the decoding performance, such as adding layers of the RBMs and utilizing search algorithms to optimize the hyper-parameters of the DBN. Additionally, optimal frequency bands associated with relevant motor imagery tasks can be further investigated to promote the overall performance of the proposed method. For the segmentation processing of the sliding window with 80% overlapping, there was a correlation between the 16 samples from the same EEG trial. This factor may influence the performance of the proposed method for the incomplete EEG classification. In the next work, similar to the study of Asensio-Cubero et al., a comparative research should be conducted by applying the proposed method to three different segmentation strategies: (1) no segmentation, by applying the proposed method directly to the whole EEG trial, (2) uniform segmentation without overlapping, and 3) segmentation with different overlapping (sliding window method) (Asensio-Cubero et al., 2011). In this study, the BCI system based on motor imagery EEG works in a synchronous way. And an asynchronous BCI system needs to be further investigated in the future work. In conclusion, the introduced decoding scheme provides an effective solution for the incomplete motor imagery EEG in the BCI system.

## Author contributions

YC, XZ, YijZ, WX, and JH conceived the conception and designed the decoding scheme for this research. YC and YZ carried out the comparative experiments, including acquisition and analysis of data for the work. YC, XZ, and YijZ interpreted the experimental results. YC drafted the manuscript. XZ, WX, JH, and YiwZ revised the manuscript.

### Conflict of interest statement

The authors declare that the research was conducted in the absence of any commercial or financial relationships that could be construed as a potential conflict of interest.
